# Transcriptional profiles of Arabidopsis stomataless mutants reveal developmental and physiological features of life in the absence of stomata

**DOI:** 10.3389/fpls.2015.00456

**Published:** 2015-06-23

**Authors:** Alberto de Marcos, Magdalena Triviño, María Luisa Pérez-Bueno, Isabel Ballesteros, Matilde Barón, Montaña Mena, Carmen Fenoll

**Affiliations:** ^1^Facultad de Ciencias Ambientales y Bioquímica, Universidad de Castilla-la ManchaToledo, Spain; ^2^Departamento de Bioquímica, Biología Celular y Molecular de Plantas, Estación Experimental del ZaidínGranada, Spain

**Keywords:** epidermis development, fluorescence imaging, *mute-3*, photosynthesis, *spch-3*, stomata, transcription factor, transcriptome

## Abstract

Loss of function of the positive stomata development regulators *SPCH* or *MUTE* in *Arabidopsis thaliana* renders stomataless plants; *spch-3* and *mute-3* mutants are extreme dwarfs, but produce cotyledons and tiny leaves, providing a system to interrogate plant life in the absence of stomata. To this end, we compared their cotyledon transcriptomes with that of wild-type plants. K-means clustering of differentially expressed genes generated four clusters: clusters 1 and 2 grouped genes commonly regulated in the mutants, while clusters 3 and 4 contained genes distinctively regulated in *mute-3*. Classification in functional categories and metabolic pathways of genes in clusters 1 and 2 suggested that both mutants had depressed secondary, nitrogen and sulfur metabolisms, while only a few photosynthesis-related genes were down-regulated. *In situ* quenching analysis of chlorophyll fluorescence revealed limited inhibition of photosynthesis. This and other fluorescence measurements matched the mutant transcriptomic features. Differential transcriptomes of both mutants were enriched in growth-related genes, including known stomata development regulators, which paralleled their epidermal phenotypes. Analysis of cluster 3 was not informative for developmental aspects of *mute-3*. Cluster 4 comprised genes differentially up−regulated in *mute−3*, 35% of which were direct targets for SPCH and may relate to the unique cell types of *mute−3*. A screen of T-DNA insertion lines in genes differentially expressed in the mutants identified a gene putatively involved in stomata development. A collection of lines for conditional overexpression of transcription factors differentially expressed in the mutants rendered distinct epidermal phenotypes, suggesting that these proteins may be novel stomatal development regulators. Thus, our transcriptome analysis represents a useful source of new genes for the study of stomata development and for characterizing physiology and growth in the absence of stomata.

## Introduction

Stomata regulate CO_2_ uptake and water loss, and are essential for cooling down plant leaves, and for pumping water and nutrients from roots to shoots through transpiration streams. They also play a pivotal role in global carbon and water cycles (Hetherington and Woodward, 2003). Although environmental conditions regulate the extent of stomata aperture, stomata operate within relatively narrow margins, which are set for their optimal function and are rarely wide open (reviewed in Dow and Bergmann, 2014; Dow et al., 2014a). Therefore, their abundance and distribution patterns are key for determining the maximum area available for gas exchange, thus impinging on plant survival and reproduction.

The adoption of *Arabidopsis thaliana* (Arabidopsis hereafter) as a model for stomatal development has produced a wealth of information regarding the genetic control of this process (Dong and Bergmann, 2010; Pillitteri and Dong, 2013). Stomata differentiation takes place gradually during organ development (Geisler and Sack, 2002) via the interplay of a genetic programme with environmental cues and results in different stomatal numbers under different conditions (Casson and Gray, 2008; Casson et al., 2009; Kang et al., 2009; Xie et al., 2010; Delgado et al., 2012; Tricker et al., 2012; Casson and Hetherington, 2014; Kumari et al., 2014). To date, the mechanisms that link environmental cues to the gene circuits that regulate stomata development remain largely unknown. Recently, the developmental response to elevated atmospheric CO_2_ was analyzed (Engineer et al., 2014). The analysis involved two carbonic anhydrases and an extracellular protease, which promotes accumulation of the signaling peptide EPF2, a repressor of stomatal development. Evidence exists for a broad intraspecific natural variation in stomatal abundance (Woodward et al., 2002). A detailed study unveiled accessions with extremely high or low values, suggesting that genetically determined stomatal abundance may have an adaptive value in natural environments (Delgado et al., 2011). In the past years, a combination of genetic, genomic, and biochemical approaches have contributed to dissecting gene circuits regulating stomata development, their abundance and patterns (Lau and Bergmann, 2012). While these studies have identified only a few positive regulators of stomata development, they describe a wealth of genes that ensure correct stomata patterns. Amongst these negative regulators of stomatal development are membrane receptors and receptor kinases (TMM and the ERECTA family), signaling peptides, and specific members of MAP kinase cascades, among others (Pillitteri and Torii, 2012; Torii, 2012; Wengier and Bergmann, 2012).

This research efforts have generated mutant or engineered Arabidopsis genotypes with various stomatal abundances and patterns, establishing correlations between reduced stomatal densities and reduced transpiration/increased water-use efficiency (Von Groll et al., 2002; Yoo et al., 2010; Chakravorty et al., 2011; Xing et al., 2011; Franks et al., 2015). Recent work has analyzed a range of genotypes with distinct stomatal abundances and patterns, determining how epidermal phenotypes impinge on physiological parameters such as stomatal conductance or CO_2_ assimilation. These studies have also highlighted the relevance of proper stomata spacing and have revealed the physiological consequences of different stomatal densities (Dow et al., 2014a,b). Microarray analysis of various genotypes has also proven useful for describing developmental aspects and for identifying new genes (Hachez et al., 2011; Pillitteri et al., 2011). Other genome-wide approaches have identified *in vivo* chromatin binding sites for SPCH (Lau et al., 2014), providing a suite of putative target genes for this transcription factor, which acts as a positive regulator of stomata development.

**Figure 1:**
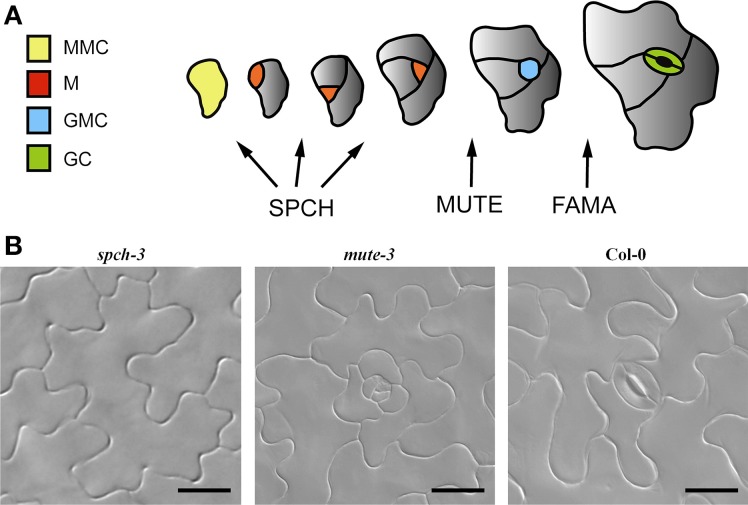
**Stomatal development in Arabidopsis. (A)** Arabidopsis stomatal development from a protodermal (Meristemoid Mother cell, MMC; yellow) involves sequential cell division and differentiation events that can be grouped into three main stages, regulated by three bHLH transcription factors: SPEECHLESS (SPCH), MUTE, and FAMA. SPCH is required for up to three asymmetric divisions that generate the cells in the stomatal lineage: meristemoids (M, red) and stomatal lineage ground cells (gray) that will eventually differentiate into pavement cells. MUTE is essential for the transition of the youngest meristemoid to a guard mother cell (GMC, blue) and FAMA controls the symmetric division of the GMC, which produces the guard cell pair (GC, green). **(B)** DIC micrographs of adaxial cotyledons showing the epidermal phenotypes of the genotypes used in this work. Loss-of-function mutations in *SPCH* (*spch-3)* prevent the initiation of stomatal lineages, while mutations in *MUTE* (*mute-3*) allow lineages to initiate and develop, but they arrest prior to stomata differentiation. Col-0 is a wild-type accession. Bar, 20 μm.

As stomata are crucial for land plants, species lacking stomata are very rare (Woodward, 1998). However, loss-of-function of positive stomatal development regulators gives rise to stomataless phenotypes. Among these positive regulators are three related basic helix-loop-helix (bHLH) transcription factors that drive entry into stomatal lineage (SPEECHLESS, SPCH; Macalister et al., 2007), transit from meristemoid to guard mother cell (GMC) (MUTE; Pillitteri et al., 2007) and terminal differentiation of GMC into paired GCs (FAMA; Ohashi-Ito and Bergmann, 2006) (Figure 1A). Loss-of-function mutations in any of these genes give rise to stomataless plants (Figure 1; reviewed by Dong and Bergmann, 2010). *spch* mutants produce only pavement cells (Macalister et al., 2007); *mute* mutants produce arrested stomatal lineages (Pillitteri et al., 2007; Triviño et al., 2013) and *fama* mutants produce caterpillar-like GMC tumors instead of forming stomata (Ohashi-Ito and Bergmann, 2006).

Although mutants lacking stomata are extreme dwarfs, their cotyledons are amenable to molecular, physiological and developmental analysis. Accessing their genome-wide transcriptional complement will provide unbiased clues as to which genes and pathways are active under the conditions imposed by their genotypes. In this work, we exploit the transcriptomes of *spch-3, mute-3*, and Col-0 cotyledons to identify the molecular trends of stomataless plants (genes similarly expressed in both mutants that had distinct epidermal cell types) and of *mute-3* (genes differentially expressed only in this mutant and potentially related to developing stomatal lineages). We made predictions from transcriptome analysis related to physiology or development and tested them functionally by *in vivo* fluorescence imaging, as well as by phenotyping loss-of-function mutants and transgenic conditional overexpressing lines.

## Materials and methods

### Plant material and growth conditions

*Arabidopsis thaliana* L. (Heyn) ecotype Columbia-0 and the mutant line *spch-3* (Macalister et al., 2007) were obtained from NASC (accessions N1092 and SAIL_36_B06, respectively); *mute-3* is described in Triviño et al. (2013). Transgenic TRANSPLANTA lines are described in Coego et al. (2014). T-DNA insertion lines were purchased from NASC and homozygous lines were generated and genotyped (Table S1). All lines were in a Col-0 background.

For *in vitro* growth, sterile seeds were sown in 1X MS + 1% sucrose plates and kept at 4°C in the dark for 48 h. Seedlings were grown in chambers with a 16 h photoperiod, 21°C, 60% relative humidity and 70 μmol photon cm-2 s-1, as described in Delgado et al. (2011).

### RNA extraction and microarray hybridization

RNA was extracted from 30 cotyledons collected 21 days post germination (dpg). Mutant genotypes were maintained in heterozygous stocks and segregating homozygous seedlings were identified by microscopic inspection. For each mutant seedling, one cotyledon was phenotyped and the other frozen in liquid nitrogen. Nine independent samples/genotype were collected and pooled into three biological replicates for RNA extraction.

RNA was extracted with Trizol/RNeasy as described in Triviño et al. (2013). RNA quality was tested with an Agilent 2100 Bioanalyzer (Brolingen). Sample preparation and hybridization with ATH1 GeneChip (Affymetrix) was performed at the Centro Nacional de Biotecnología (Madrid). Three independent biological samples were used to hybridize each of the three slides used. The original hybridization results were deposited in ArrayExpress (accession number E-MTAB-3416).

### Transcriptome analyses

Background correction, normalization and expression data averaging following optical reading of GeneChips with a 3000 7G scanner (Affymetrix), as well as conversion to.CEL files, was performed by robust multichip analysis (RMA) (Irizarry et al., 2003). Gene expression values were adjusted to a linear model to apply a contrast by Student's *t*-test using an empirical Bayes analysis (LIMMA, Smyth, 2004) and to obtain *p*-values. Benjamini and Hochberg's (1995) method was used to correct for false discovery rate (FDR). FDR adjusted *p*-values < 0.05 and fold changes between samples > ± 2 were considered significant. Transcripts absent in all genotypes were eliminated according to the MAS5 algorithm (Hubbell et al., 2002). Transcripts detected in at least two of the three replicates were considered “present.”

Genes differentially expressed (DE) among genotypes were obtained using the VENNY software program (Oliveros, 2007). Functional classification of Venn diagram intersections was performed with MapMan 3.0.0 (Thimm et al., 2004) for **Figure 5**. Gene Ontology-based software AgriGO (Du et al., 2010) was used for Supplementary Figures S1, S2 and S3 using TAIR 10. Clustering was made using a k-means clustering algorithm (*k* = 4; covariance as a distance method) with Genesis software (Sturn et al., 2002).

### Real time PCR

Expression levels were estimated by quantitative RT-PCR, using cDNA obtained from the RNA samples used for microarray hybridization, with the High-Capacity cDNA Archive Kit (Applied Biosystems); qPCR was performed on a LightCycler® 480 II Real-Time PCR instrument (Roche), using the Maxima SYBR Green qPCR Master Mix (Thermo Scientific) and the primer sets listed in Table S2. Three biological replicates were analyzed per genotype. C_T_ values and relative expression changes were obtained with the LightCycler® 480 software version 1.5 (Roche) and determined by the efficiency method, where fold change is calculated as E^CtT(C)−CtT(S)^_T_ × E^CtR(S)−CtR(C)^_R_. *UBQ10* (At4g05320) and *ACT2* (At3g18780) were the reference genes.

### β-estradiol treatments

Phenotypic effects of TF overexpression in the TRANSPLANTA lines (Coego et al., 2014) were tested by germinating seeds on MS containing 10 μM 17-β-estradiol (E8875, Sigma) and epidermal inspection 6 days later. At least five plants per line were examined. Lines showing phenotypes were tested at least twice.

### Microscopy

Epidermal phenotypes were determined by DIC microscopy, as described in Delgado et al. (2011) and Triviño et al. (2013).

### Imaging of chlorophyll-fluorescence kinetics

Fluorescence kinetics were recorded according to Granum et al. (2015) using an Open FluorCam FC 800-O (Photon Systems Instruments, Brno, Czech Republic). After 30 min of dark-adaptation, plants were illuminated at 450 μmol photon cm^−2^ s^−1^ for 10 min, followed by a 10 min relaxation period in the dark. Saturating pulses of 2000 μmol photon cm^−2^ s^−1^ and 1 s long were given 10 s after the beginning of each period, and then every 2 min. After each excitation and relaxation period, a steady-state was reached.

Maximum quantum efficiency of PSII was calculated as F_V_/F_M_ (1 – F_0_/F_M_) where F_0_ and F_M_ were the minimum and maximum fluorescence in the dark-adapted state, respectively. The quantum yield of PSII (Φ_PSII_) was calculated as 1 – F_S_/F_M_', where F_S_ and F_M_' were the fluorescence before and during saturating pulses in the light-adapted state, respectively. Non-photochemical quenching (NPQ) was calculated as F_M_/F_M_' – 1. Each experiment was repeated four times with similar results; a total of 10–20 plants/genotypes were sampled per experiment.

### Multicolor fluorescence imaging

Multicolor fluorescence imaging was carried out on the adaxial side of the leaves using an Open FluorCam FC 800-O (Photon Systems Instruments, Brno, Czech Republic). The imaging in blue (F440), green (F520), and red (F680) regions of the spectrum was acquired sequentially for each sample, as described by Granum et al. (2015). Experiments were repeated four times with similar results; a total of 10–20 plants/genotype were sampled per experiment.

### Pigment composition

The content on chlorophylls and xanthophylls plus carotenoids was determined in 90% methanol extracts, as per Lichtenthaler and Buschmann (2001).

## Results and discussion

### Transcriptomes of stomataless mutants

To investigate gene expression profiles associated with the developmental and physiological features of stomataless plants, a transcriptomic analysis was performed for two mutants lacking stomata, i.e., *spch-3* and *mute-3* and the wild-type, stomata-bearing Col-0 accession. RNA from 21 dpg cotyledons was used to hybridize Affymetrix ATH1 microarrays (see Materials and Methods).

**Figure 2:**
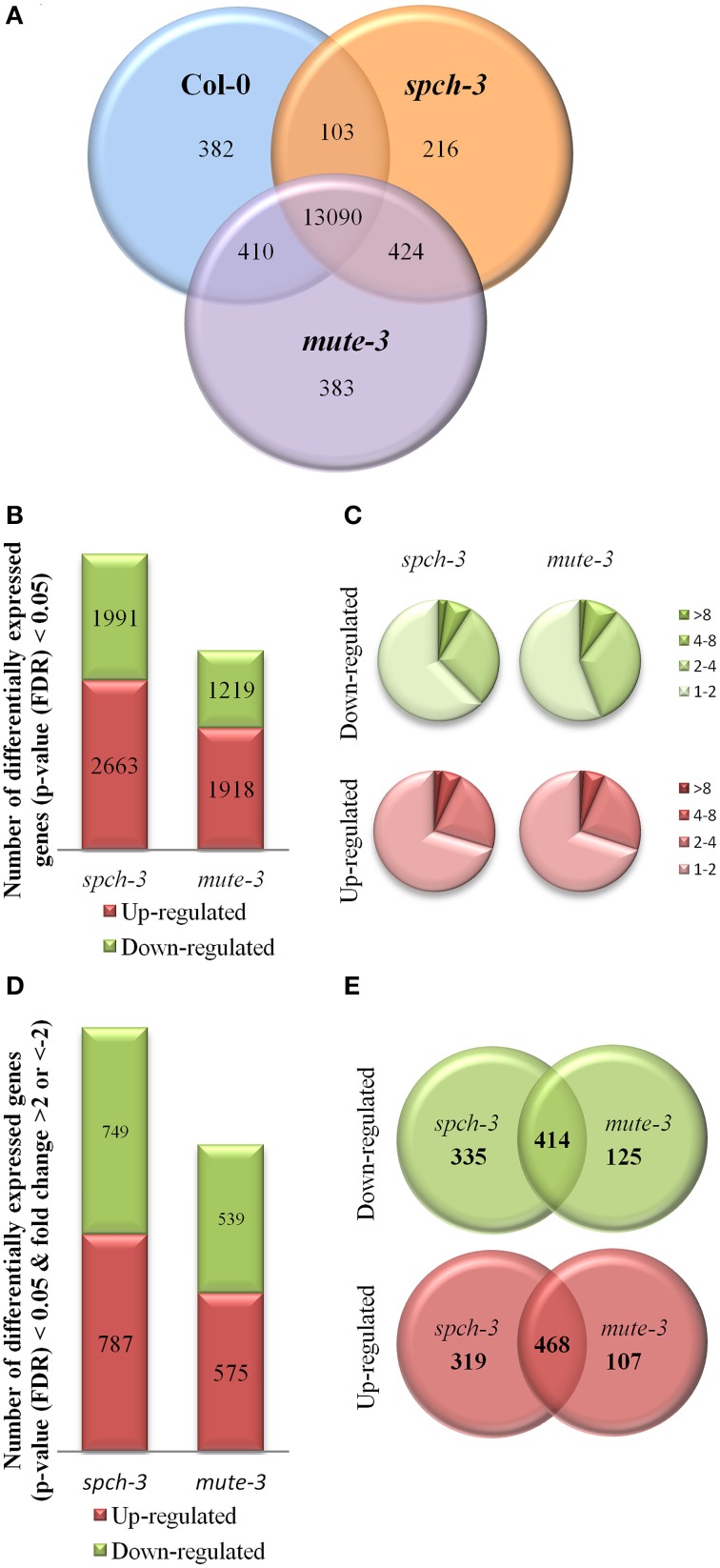
**Comparative transcriptomic analysis of *spch-3*, *mute-3*, and Col-0 cotyledons. (A)** Venn diagram showing the intersection of all genes expressed in each genotype (MAS5-present call in at least two biological replicates in the array). **(B)** Genes differentially expressed in each mutant with respect to Col-0 (LIMMA *FDR* < 0.05) and **(C)** their distribution by fold-change difference. **(D)** Genes differentially expressed with a fold-change >2 or <–2 in each mutant, compared to Col-0 and **(E)** Venn diagrams showing their intersections. Transcripts correspond to three independent replicates of RNA extracted from 21 dpg cotyledons.

Transcriptomes were first analyzed for present/absent transcripts, using the MAS5.0 algorithm to set the detection threshold in the Affymetrix chip (Hubbell et al., 2002) and selecting transcripts present in at least two biological replicates. Approximately 14,000 genes were expressed in each genotype (Col-0: 13,985; *mute-3*: 14,307; *spch-3:* 13,833), most of which (ca. 13,000) were common to all genotypes. For almost 2000 genes, transcripts were present in only one or two genotypes. Genes exclusively expressed in one genotype were 216 in *spch-3*, 383 in *mute-3* and 382 in Col-0 (Figure 2A).

To determine if the experimental design allowed for detecting genotype-characteristic transcripts, we examined the expression of genes previously involved in stomatal development. Col-0 cotyledons had only pavement cells and stomata, *mute-3* showed pavement cells and developing stomatal lineages, including arrested meristemoids and *spch-3* had exclusively pavement cells. All genotypes accumulated transcripts for *ERECTA, ERL1*, *YDA, MKK4/5, MPK3/6*, *STOMAGEN* and *SCREAM/ICE1*, and *SCREAM2*, whose expression is not exclusive of developing stomatal lineages or specific cell types. In contrast, the genotypes differed in transcripts specific for particular stomatal lineage cell types. For instance, only *mute-3* expressed the immature lineage markers *MUTE*, *EPF2*, and *TMM*, and only Col-0 expressed the guard cell marker gene *FAMA*. As expected, transcripts for all these genes were absent in *spch-3*. Thus, the observed transcriptional profiles matched the epidermal phenotypes, even for genes expressed in cell types or stages with a very small contribution to all cells represented in the samples. These results strongly support the experimental approach, as it discriminates genotype-specific, low-abundance transcripts. Consequently, these transcriptomes should also contain transcripts from undescribed genes expressed preferentially/exclusively in each of the three genotypes.

Next, pairwise comparisons of gene expression levels were conducted using LIMMA (Smyth, 2004) for a FDR < 0.05 (Figure 2B). The results showed that the number of differentially expressed (DE) genes with respect to Col-0 was higher in *spch-3* than in *mute-3*. The amplitude of DE (fold-change values) with Col-0 ranged between −32 and 53 for *mute-3* (corresponding to a plant-specific protein of unknown function encoded by At1g64360 and the transcription factor PISTILLATA), and between −63 and 209 for *spch-3* (for transcripts encoding the cell wall protein ECS1 and PISTILLATA, respectively). In both mutants, approximately 8% of the DE genes had absolute fold-change values >4 and around 27% presented values between 2 and 4 (Figure 2C). Genes DE, with an absolute fold-change >2, were 1114 in *mute-3* and 1536 in *spch-3* (Figure 2D and Table S3); only a fraction of these genes were regulated in a similar manner in the two mutants (Figure 2E).

**Figure 3:**
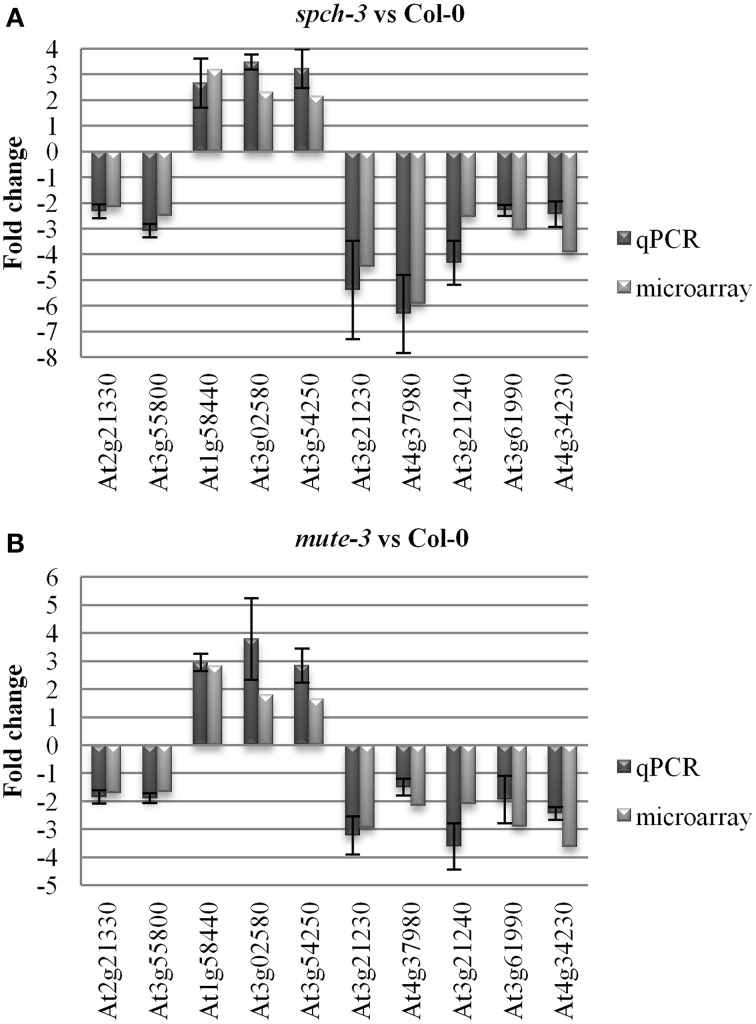
**Quantitative PCR for microarray validation**. Arrays were validated by quantitative RT-PCR for 10 genes selected from the list in Table S3. *UBIQUITIN10* and *ACTIN2* served as reference genes. The fold change of the qPCR was determined by the efficiency method (E^CtT(C)-CtT(S)^_T_ × E^CtR(S)-CtR(C)^_R_). The results of qPCR for *spch-3* **(A)** and *mute-3* **(B)** respect to Col-0 were averaged from three independent experiments, with the error bar indicating the standard error of the mean.

Relative expression levels for a panel of genes in the three genotypes was further tested by qPCR, selecting 10 genes that in the microarray comparisons were similarly up- or down-regulated in both mutants, compared to Col-0. The results (Figure 3) showed that for the 10 genes, expression tendencies were the same as estimated by the two independent methods.

A previous microarray study involving *spch-3* and other stomatal mutants (Pillitteri et al., 2011) was designed to minimize physiological differences among genotypes, while the present work aimed at stressing such differences. Thus, when compared, these studies attempt to answer different questions and are not comparable. In the present analysis, the high number of DE genes in the mutants might similarly reflect profound differences in growth and physiology among wild-type and stomataless mutant plants, and not merely their epidermal phenotypes, which were different in the two mutants. To our knowledge, the present study is the first to offer a description of a *mute* mutant transcriptome.

### General transcriptomic features common to stomataless mutants

**Figure 4:**
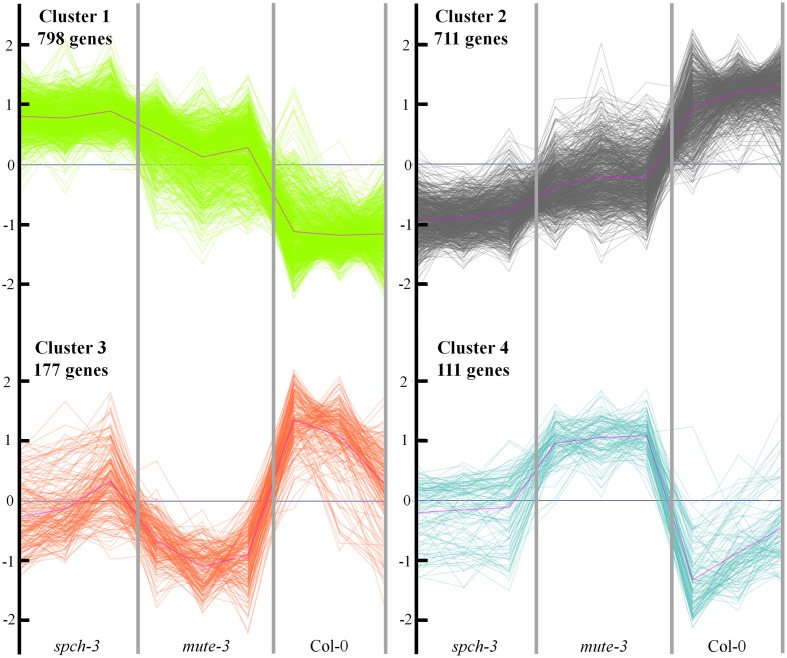
**Cluster analysis of expression profiles for differentially expressed genes in the three genotypes**. A total of 1797 genes significantly (*FDR* < 0.05) up- (>2 fold) or down (<–2 fold) regulated in any of the mutants, with respect to Col-0 or between the two mutants, was selected. The normalized expression values were used to perform a k-means clustering for *k* = 4 (see Methods). The Y-axis shows normalized fold-changes in gene expression and the X-axis indicates each genotype, with the three biological replicates independently represented. Genes classified in each cluster are listed in Table S4.

We expected genes DE in *spch-3* or in *mute-3*, with respect to Col-0, to include those related to the physiology of stomataless plants and to genotype- or cell type-specific trends. To characterize these gene expression patterns, we performed a k-means cluster analysis, using normalized values of the three biological replicates for each genotype. For the analysis, 1797 genes were selected, based on whether they were significantly (*FDR* < 0.05) up- (>2 fold) or down-regulated (<–2 fold) in any of the mutants, with respect to either Col-0 or between the two mutants. Z-score transformations of the log_2_ expression values were used for a k-means clustering (*k* = 4) (Figure 4). Genes classified in each cluster are listed in Table S4. Clusters 1 and 2 include, respectively, genes with higher or lower expression in the two stomataless genotypes than in Col-0. Clusters 3 and 4 include genes with a lower or higher expression in *mute-3*, with respect to the other genotypes, thereby representing expression patterns characteristic of *mute-3*. These genes may relate to epidermal differences of *mute-3* with *spch-3* and Col-0 (Figure 1B), and they are described in the sections devoted to development.

To identify biological processes similarly altered in both mutants, we applied a gene set enrichment analysis to clusters 1 and 2 (GO database through AgriGO software). Cluster 1 analysis revealed increased transcription of genes classified in several functional categories (Supplementary Figure S1): *lipid localization*, *regulation of cell size* and *developmental growth*. These categories include important growth regulators like *PIN3*, *COBRA*, and *BRI1*. There is also an enriched group of categories involving *carbohydrate metabolic processes* like polysaccharide and glucan biosynthesis (mostly related to cell wall; see below). In cluster 2, genes with decreased expression in stomataless mutants belonged to three overrepresented categories (Supplementary Figure S2), with *cellular nitrogen compound metabolic process* appearing as a hub. Genes involved in the biosynthesis of glucosinolates and glycosinolates were mostly depressed, as were the master regulators of these pathways, MYB28, MYB29, and MYB34 (Frerigmann and Gigolashvili, 2014). Down-regulation of genes related to *response to stimulus*, e.g., chemicals, abiotic cues, or water deprivation was also patent.

**Figure 5:**
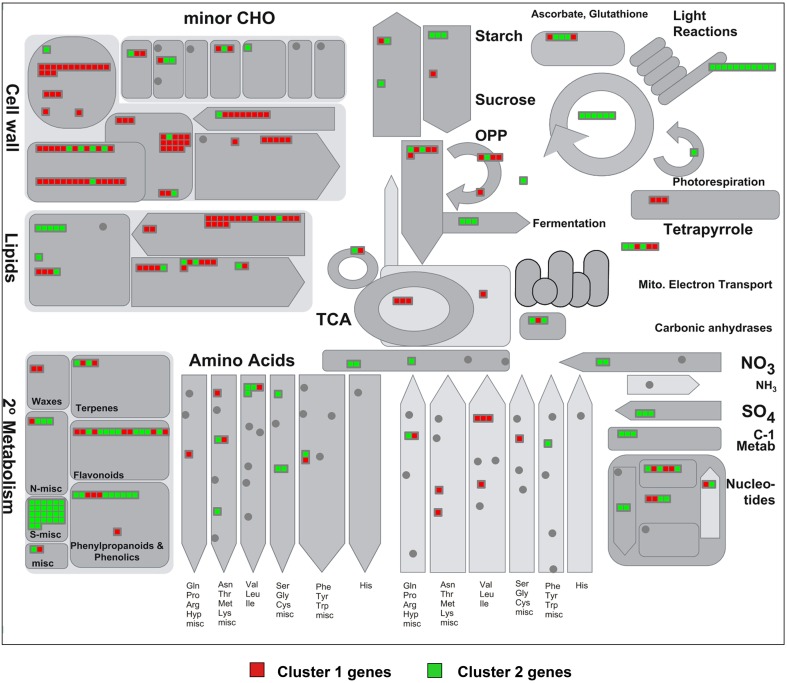
**MapMan visualization of the differences in metabolism-related gene expression between stomataless mutants and wild-type plants**. Differentially expressed metabolic genes classified in clusters 1 and 2 (Figure 4), and thus similarly regulated in both mutants, were analyzed using MapMan software. Each square represents a gene and displays a qualitative color code: red for genes up-regulated (cluster 1) and green for those down-regulated (cluster 2) in the mutants with respect to Col-0. Note the pathways with predominantly up-regulated (mostly red) and down-regulated (mostly green) genes.

We then identified the MapMan metabolic pathways altered in the two mutants based on the gene sets of clusters 1 and 2. Figure 5 represents the common up-regulated (cluster 1) genes in red and in green, common down-regulated (cluster 2) genes (see Figure 4 and Table S4). Almost all DE cell wall-related transcripts cataloged in MapMan were up-regulated in *spch-3* and *mute-3*; they encoded proteins involved in the biosynthesis of precursors, cellulose and hemicelluloses, arabinogalactan and proline-rich wall proteins, extensins, expansins, pectin esterases, and other cell wall-remodeling proteins. These patterns may reflect a potential for growth in the mutants, while Col-0 cotyledons were terminally expanded, according to the biological processes predominant in both mutants (Figures S1, S2). Lipid metabolism-related DE genes were mostly up-regulated. They included 18 genes that encode enzymes involved in fatty-acid synthesis and elongation, e.g., acyl-carrier proteins, acetyl-CoA carboxylases, ketoacyl-ACP synthases and reductases, desaturases, and others. Most lipases were up-regulated, including the membrane-anchored glycerophosphoryl diester phosphodiesterase-like proteins SHAVEN-3 and SHV3-LIKE 1, involved in cell wall deposition and root hair and trichome differentiation (Hayashi et al., 2008). Both genes are expressed during stomatal development, according to the Arabidopsis eFP Browser (Winter et al., 2007); furthermore, a double *sha3;slv1* mutant displayed enlarged guard cells (Hayashi et al., 2008), suggesting that both participate in stomatal-lineage cell differentiation. Some transcripts for enzymes related to sphingolipid synthesis were also up-regulated in both mutants. In contrast, the five DE genes in *phospholipid synthesis* were down-regulated.

Overall, the transcriptomes of stomataless mutants suggest a depressed secondary metabolism and dampened mechanisms for stress responses, while maintaining growth-related processes. These trends are hardly surprising, considering that CO_2_ acquisition is presumably very limited in plants with no stomata. Their extremely slow but sustained growth may as a result monopolize most carbon and energy resources, preventing major investments in non-essential processes.

Genes similarly regulated in both mutants, as compared to Col-0 classified in the remaining metabolic pathways, were mostly down-regulated and are discussed below.

### Photosynthesis in stomataless mutants

The number of photosynthetic genes DE in both mutants that met the filters of fold-change and statistical significance set for the clustering was surprisingly low. However, most of the down-regulated genes code for crucial proteins, whose depletion should impair photosynthesis. All significant changes in PSII corresponded to repressed genes and included those coding for PsbB, PSBTc, PsbN, PsbQ-LIKE 1, PsbQ-LIKE 2, and PsbP-like protein 2. Other genes repressed with various levels of significance and fold-changes were nuclear or chloroplast genes coding for PSII protein subunits, such as PsbO2 (a regulating subunit in the oxygen-evolving complex), PsbH, PsbA (core protein D1), and PsbC (core chlorophyll-binding protein). The gene encoding the PSI reaction center subunit PsaA was repressed, as were those for five NADH-dehydrogenase subunits (NDH-O, N, D, H, and J). Cytochrome f was down-regulated, as was *IMMUTANS*, which encodes a chloroplast alternative oxidase (Wu et al., 1999). ATPase subunits ATPB and ATPF transcripts were decreased. Regarding CO_2_ assimilation, several Calvin cycle genes were repressed, most remarkably the chloroplast gene *rbcL* encoding the large subunit of RubisCO, but also genes encoding fructose bis-phosphate aldolase (FBA1), several phosphoglycerate kinases and a sedoheptulose bisphosphatase. A photorespiration-related glycolate oxidase was also down-regulated.

This transcriptional profile shared by the two mutants suggests impaired photosynthetic light reactions, low production of reducing power and ATP and depressed carbon assimilation, as expected of plants lacking stomata. However, chloroplasts do not seem dysfunctional in these mutants. Some genes related to tetrapyrrole metabolism, as well as *GUN4*, which couples nuclear gene transcription to chloroplast status (Larkin et al., 2003), were up-regulated. Several antenna protein subunits were also up-regulated, although with differences not always statistically significant. Therefore, the down-regulation in *spch-3* and *mute-3* of photosynthesis-related genes seems to be specific and not the result of general chloroplast dysfunctions. It is also possible that many genes in this category did not change their transcription, but that the corresponding proteins were more or less abundant or active in the mutants due to differences in post-transcriptional regulation.

**Figure 6:**
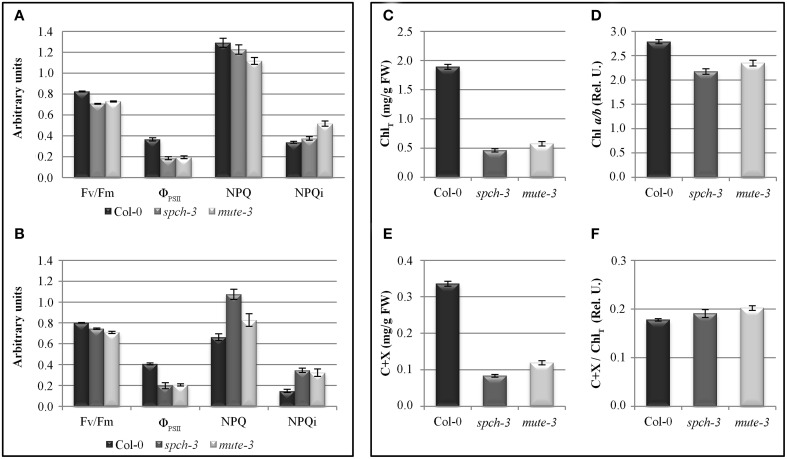
**Photosynthetic parameters and pigments in wild-type and stomataless mutants**. Left panel: photosynthesis parameters calculated by quenching analysis of chlorophyll fluorescence in 21-day-old plants **(A)** and after 4 days acclimation to soil **(B)**. Right panel: total chlorophyll content (Chl_T_ **(C)**), Chl *a/b* ratio **(D)**, carotenoids and xanthophylls (C+X **(E)**), and carotenoids and xanthophylls over total chlorophyll content **(F)** in Col-0, *spch-3*, and *mute-3* plants after adaptation to soil. Error bars are SE. All pairwise differences were statistically significant in a Student *t*-test (*N* = 10; *T*-test *p*-value < 0.038), except NPQ differences between *spch-3* and Col-0 in **(A)**.

The physiological impact of the observed transcriptional changes for photosynthesis-related genes was examined *in vivo* by quenching analysis of chlorophyll fluorescence. Kinetic analyses of the red fluorescence emitted by chlorophyll estimated photosynthetic efficiency, as well as mechanisms of energy dissipation indicative of stress. For these experiments, we tested plants grown *in vitro* for 21 days (as in the microarray experiments) or plants adapted to soil for 4 extra days. Figures 6A,B show that *spch-3* and *mute-3* displayed a statistically significant decrease in Fv/Fm compared to Col-0, indicating a dysfunction of the photosynthetic machinery. Both mutants also showed low values for PSII quantum yield (Φ_PSII_) relative to wild-type plants, suggesting an inhibition of photosynthetic electron transport. The decrease in Φ_PSII_ was associated with a larger capacity for energy dissipation (measured as non-photochemical quenching, NPQ) typical of stress conditions and a higher proclivity for photoinhibition, as shown by the higher irreversible NPQ (NPQi). We observed similar trends in 7 dpg cotyledons, although only differences in Fv/Fm were statistically significant (not shown).

These results are consistent with a retroinhibition of the thylakoid electron transport chain, perhaps due to limiting concentrations of internal CO_2_, which inhibit the Calvin cycle. The strong down-regulation of *rbcL* transcripts and the repression of genes coding for subunits of PSII and other complexes of the electron transport chain also support this interpretation.

**Figure 7:**
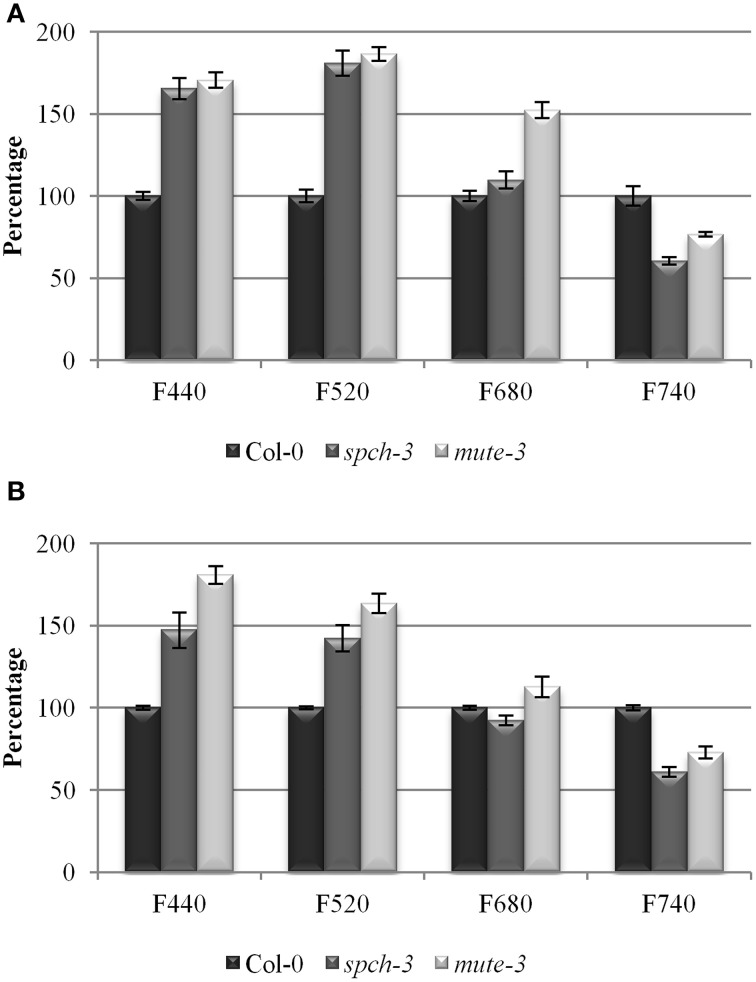
**Multicolor fluorescence in the three genotypes**. Relative fluorescence as a percentage of Col-0 in the blue (F440), green (F520), red (F680), and far red (F740) regions of the spectrum for 21-day-old plants **(A)** and after 4 days acclimation to soil **(B)**. Error bars are SE. All pairwise differences were statistically significant in a Student *t*-test (*N* = 10; *T*-test *p*-value = 3.74 E-07).

Relative content of chlorophylls (Chl) *a* and *b* and accessory pigments (carotenoids and xanthophylls, C + X) were measured in leaf extracts (Figures 6C–F). As expected due to their pale phenotypes, both mutants had a reduced content of all photosynthetic pigments on a fresh weight basis, compared to Col-0. Chl *a/b* ratios were significantly reduced, while C+X/Chl ratios increased. These results suggest that pigment composition of the photosynthetic complexes I and II, most of which reside in their antenna complexes, is altered in the mutants. The decrease in Chl *a/b* ratio is compatible with an increase in the proportion of antenna complexes/reaction centers, suggested by the transcriptomic data. DE transcripts in the category *antenna proteins* had many up-regulated genes (six genes vs. one repressed, all significant and close to the fold-change threshold). The opposite occurred for *reaction centres*, with five down-regulated genes (all from PSII). The low chlorophyll content in the mutants explains the decrease in the intensity of fluorescence observed in the far-red region (F740) (Figure 7). Alterations in the intensity of red fluorescence (F680) may stem from changes in the Chl *a/b* ratio in the mutants. Thus, both chlorophyll content and photosynthetic efficiency estimations were those expected from the transcriptomic profiling of the two stomataless mutants and suggested depressed, albeit not null, photosynthetic activity.

In the present work, plants grew in a sucrose-supplemented medium (see Materials and Methods). Sucrose appears to be taken-up and used by the mutants, as suggested by increased transcripts for the phloem unloading sucrose transporter ATSUT2 (Ayre, 2011) and the degradative invertase CINV1 (Barratt et al., 2009). The activation of glycolysis, suggested by the up-regulation of genes coding for plastidic and cytosolic pyruvate kinase subunits, as well as other enzymes from the PPO and TCA cycles may contribute to plant growth. Transcripts for fermentative enzymes were, however, down-regulated. The fact that *spch-3* and *mute-3* can to some extent grow in soil also indicates that they do perform photosynthesis, perhaps helped by more active carbonic anhydrases, as well as a modified cuticle that might be more permeable to CO_2_. However, growth was much more extensive (notably for *mute-3*) in sucrose-containing medium.

The dwarf *spch-3* and *mute-3* phenotypes, when growing on sucrose, may reflect other physiological constraints for growth without stomata. These possible constraints include, e.g., limited O_2_, depressed transpiration hindering nutrient and water root uptake, as well as evaporative cooling or the accumulation of volatile hormones or metabolites. In *spch-3*, there are very limited epidermal cell divisions (as stomatal lineages that contribute to epidermal cell numbers are absent). This fact might impose another constraint for growth and explain why dwarfism is more drastic in s*pch-3* than in *mute-3*.

### Nitrogen, sulfur, and secondary metabolisms

DE genes related to the assimilation of sulfur (such as those coding for one APS and two APS-Kinases) and nitrogen (those coding for GLU and GLN-synthases and two nitrate transporters) were all down-regulated in *spch-3* and *mute*-*3*. Both mutants displayed repression of all 26 DE genes involved in glucosinolate metabolism, 19 of which are in the biosynthetic pathway. Glucosinolates are nitrogen and sulfur-rich secondary metabolites, whose accumulation depends on sulfur status (Falk et al., 2007). Previous metabolite and transcript profiling revealed coordinated repression of most glucosinolate-related genes in response to sulfate limitation (Hirai et al., 2005). Also repressed were three out of the four DE genes related to N-containing alkaloids. Given the depressed photosynthetic capacity, which might limit reducing power and ATP needed for N and S metabolisms, this behavior was not surprising. In contrast, *EXORDIUM-LIKE* (*EXL*) genes *EXO*, *EXL1*, *EXL3*, *EXL4*, and *EXL5*, which promote growth during low C and energy supply (Schroder et al., 2011) were up-regulated. Also induced was *ASN1*, whose overexpression increases plant fitness under N-limiting conditions (Lam et al., 2003) and is triggered by sugar starvation (Baena-Gonzalez et al., 2007) and photosynthesis inhibitors (Fujiki et al., 2001). These trends suggest acclimation of stomataless mutants to low carbon, reduced intermediate metabolites and energy-limiting growth conditions. DE genes related to starch metabolism (two degradative amylases and one starch synthase) were down-regulated; sucrose synthesis was also depressed, while CINV1, a cytosolic invertase related to sucrose degradation, was induced.

Most DE genes in the phenylpropanoid biosynthetic pathway such as those involved in lignin and lignane synthesis (*4CL5*, *4CL2*, *ELI3*-1, *OMTF3* y *CAD5*) were repressed, with the exception of the laccase-encoding gene *LAC8*. Flavonoid-related genes showed heterogeneous behavior. While most genes related to secondary metabolism were repressed in the mutants, some were up-regulated, notably some involved in the biosynthesis of chalcones, isoflavones, or anthocianins. For instance, a putative isoflavone reductase (At1g19540) showed strong induction. Many sterol/brassinosteroids biosynthetic genes, such as *SQE1*, *SMT3*, and *DWF7* were up-regulated. The mevalonate route, involved in carotenoid synthesis, showed induced genes, notably those encoding two mevalonate diphosphate decarboxylases, which promote accumulation of the carotenoid precursor isopentenil pirophosphate. To determine if the mutants were accumulating some end products of these pathways, we performed multicolor fluorescence analysis and found that the two mutants had an increased blue and green autofluorescence (Figure 7).

According to the transcriptomic data, the compounds responsible for this increase may be carotenoids and anthocyanins, and perhaps chalcones. Although phenols also fluoresce in this spectrum, their biosynthetic pathways are down-regulated and as such, they are improbable contributors to the observed fluorescence increases. The notion that the mutants differentially accumulate photoprotective compounds fits with their accessory photosynthetic pigment composition (Figures 6E,F), as well as their high NPQ and NPQi values (Figures 6A,B). An acclimation to the photoinhibitory conditions resulting from the inhibition of photosynthesis in both mutants may explain the accumulation of auxiliary pigments. This accumulation of putative photoprotective pigments and a depressed N and S metabolism are the most notable features of a secondary metabolism in the two stomataless genotypes.

### Growth and development in the absence of stomata

In agreement with the analysis of biological processes (Figures S1 and S2), MapMan found that DE genes in clusters 1 and 2, commonly regulated in both mutants (Tables S3 and S4), appeared in several development and growth-related categories, including genes involved in cell division and expansion. For instance, genes coding for the cyclin-dependent kinase CYCP1 and a cyclin-related protein (At2g41830), both with unknown functions, were up-regulated, as was the endoreplication factor FZR2 (FIZZY-RELATED 2). Genes for DNA synthesis encoding a telomere-binding protein, several histones or a putative helicase, as well as most genes encoding cytoskeleton and vesicle transport proteins also showed induction. The transcript with the highest induction in both mutants corresponded to *PISTILLATA*. Initially described as a flower-specific transcription factor, recent data available at the eFP Browser database (Winter et al., 2007) indicate that *PISTILLATA* is also expressed in other developmental contexts. These include the quiescent centre, the cellularized seed endosperm, root xylem pole pericycle cells in NPA-treated seedlings, endodermis and columella/root cap under low pH and trichomes of *gl3-sst* mutants. In 5-day-old *spch-3* seedlings, Pillitteri et al. (2011) also found abundant *PISTILLATA* transcripts. The reasons for its over-accumulation in *spch-3* and *mute-3* and its possible consequences remain unexplained.

Next, genes in clusters 3 and 4 were analyzed. These were DE in *mute-3* respect to Col-0 and *spch-3*, and may be characteristic of an epidermis containing developing stomatal lineages absent in the two other genotypes. Using gene set enrichment analysis (GO database through AgriGO software) we identified overrepresented biological processes. Inspection of cluster 3 revealed features similar to cluster 2; this was not informative regarding developmental aspects of *mute-3*. In cluster 4 (Supplementary Figure S3), as expected, very few genes corresponded to metabolic pathways, stress or cell wall, as *mute-3* shared most of them with *spch-3*. Both mutants also shared most of the DE genes classified in the category *Development* (as described above; Figures S1 and S2), with some notable exceptions: *TMM*, a marker specific for developing stomatal lineages (Nadeau and Sack, 2002) was present in cluster 4, as was *PDF1*, specific for the developing epidermis (Abe et al., 1999). Several *RNA*-related genes were present in this cluster, including *SCZ*, a meristemoid-specific transcription factor in aerial organs (Pillitteri et al., 2011). Previous work (Pillitteri et al., 2011) using a severe “meristemoid-only” double mutant (*scrm-D;mute*), which had one cell type similar to those present in *mute-3*, overlapped only in 24 transcripts with cluster 4. In addition to the important epidermal differences between *mute-3* and *scrm-D;mute*, growth conditions and age differed between Pillitteri et al. (2011) and the present work; hence, the limited overlap of the two transcriptomes was not surprising. We also investigated whether cluster 4 might include genes previously identified as putatively regulated by SPCH (Lau et al., 2014), since SPCH is necessary for the development of the stomatal lineages present in *mute-3*. For doing so, we compared the 1274 high-confidence SPCH targets represented in the ATH1 array with the 111 genes classified in cluster 4. Results identified 36.9% (41) of the genes characteristic of *mute-3* as putative SPCH targets, a proportion much higher than the expected 5.6% by random hits. Therefore, cluster 4 showed highly significant enrichment in genes that might be under direct SPCH regulation.

In this analysis, we also found genes not previously involved in stomatal development. Some examples are *BAM2*, encoding a CLAVATA1-related receptor kinase-like protein, which is needed for cell fate specification in meristems (Deyoung and Clark, 2008), as well as *CLE17*, which encodes a putative extracellular peptide similar to CLV3 (Meng and Feldman, 2010). Also induced in *mute-3* were *TCP3*, involved in heterochronic leaf development (Koyama et al., 2007) and *NAC35/LOV1* (*LONG VEGETATIVE PHASE1*). We found that *LOV1* co-expressed with the GC genes *FAMA*, *KAT2*, and *MYB60* (in ATTED, Obayashi et al., 2007), and was expressed in GCs (Leonhardt et al., 2004). *EDA17*, which will be described below, was found in this cluster. These data enforce the potential of cluster 4 as a source of novel genes involved in stomatal lineage development.

### Loss-of-function of genes differentially expressed in the mutants

We selected a set of 24 DE genes in *spch-3* and/or *mute-3* compared to Col-0 to inspect the phenotypic effects of their loss-of-function (Table S1). For three genes (*LOV1*, *SOL1*, and *PI*), promoter::reporter fusions were also tested in wild-type plants. Cotyledons of 5 and 10 dpg seedlings from homozygous T-DNA insertion lines were inspected for stomatal and pavement cell morphology, size, and spatial pattern. Only one of these mutants, carrying a T-DNA insertion in *EDA17*, had an epidermal phenotype; its stomatal index (proportion of epidermal cells that are stomata) was reduced to half that of Col-0 (Supplementary Figure S4). *EDA17* was originally named *HOTHEAD* (*HTH*) by Krolikowski et al. (2003), because loss-of function mutants in this locus showed floral organ fusion. The line we used (SALK_024611) carried an insertion in the middle of the predicted third exon (Supplementary Figure S4) and was reported by Peng et al. (2006) as displaying the *hth* phenotype; therefore, it is considered a loss-of-function mutant. In our seed stock, we observed the reported floral organ fusion phenotype and the reduced fertility that characterizes *hth* mutants.

As *EDA17* transcripts accumulate specifically in *mute-3* and not in Col-0 or *spch-3*, *EDA17* may be associated with developing stomatal lineage cell types. This hypothesis is supported by differential accumulation of *EDA17* transcripts in the “only meristemoids” *scrm-D;mute* mutant (Pillitteri et al., 2011). EDA17 is involved in the biosynthesis of cuticular, very long-chain fatty acids (Kurdyukov et al., 2006). Since other cuticle- and epidermal wax-defective mutants display stomata pattern defects (Holroyd et al., 2002), further work on *EDA17* may strengthen the intriguing connection between cuticle composition and stomata development.

### Conditional overexpression of genes differentially expressed in *spch-3* and/or *mute-3*

**Figure 8:**
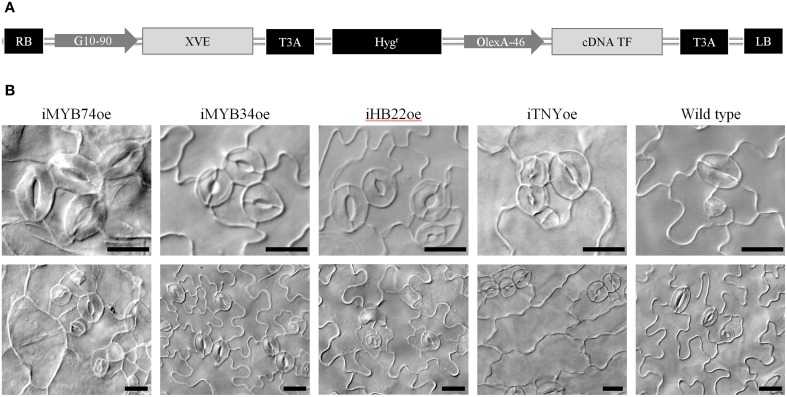
**Conditional overexpression of transcription factors differentially expressed in *spch-3* or *mute-3* transcriptomes. (A)** Diagram of the construct on pMDC7 used for generating the TRANSPLANTA lines for β-estradiol-inducible overexpression. LB, RB: left and right T-DNA borders. G10-90: artificial constitutive promoter. XVE: chimeric transcription factor activated by β-estradiol. OlexA-46: promoter recognized by activated XVE. cDNA TF: coding sequence for the different transcription factors. T3A: sequence for gateway cloning. For more details, see Coego et al. (2014). **(B)** Phenotypic effects of overexpression for selected TF. DIC micrographs of cotyledon abaxial epidermis in seedlings of the indicated genotypes (iTFoe and wild-type) grown for 6 days in β-estradiol plates. For each genotype, two representative fields are shown. Bar, 20 μm.

The suit of genes DE in *spch-3* and/or *mute-3* includes 158 transcription factors (TFs) listed in the Agris and/or PlantTFDB databases (Yilmaz et al., 2011; Jin et al., 2014), which are candidates for playing key roles in the developmental and metabolic phenotypes of the mutants. Several of the T-DNA lines we inspected corresponded to TFs; however, their loss-of-function rendered no epidermal phenotypes. Arabidopsis TFs often belong to gene families with partially redundant members; it is therefore common that loss-of-function alleles fail to provide informative phenotypes and their ectopic overexpression is used instead to hint at their putative roles. A drawback of this strategy is that it often renders deleterious phenotypes, including early lethality, which conditional overexpression systems can overcome. To examine the possible involvement of TF that were DE in the mutants, we inspected 322 transgenic lines that conditionally overexpressed 128 different TFs (Table S5). Among them, 194 lines corresponded to 76 TFs that were DE in *spch-3* and/or *mute-3*, while the remaining were their paralogues, or showed a tendency to differential expression close to statistical significance. In these lines, β-estradiol induced TF overexpression (Coego et al., 2014). Figure 8A shows a diagram of the construct used for obtaining these lines, which we termed iTFoe (inducible TF overexpressor).

For each TF-coding gene, seeds from 4 to 1 independent lines were plated on a β-estradiol-containing medium and epidermal phenotypes were inspected in 6 dpg seedlings. iFAMAoe and iMUTEoe control plants rendered the expected phenotypes (Triviño et al., 2013) with all epidermal cells transformed in guard cells or in stomata, respectively. Overexpression of 19 TFs lead to abnormal epidermal phenotypes in all or some of the independent lines. The most recurrent effects were on stomatal density (number of stomata per area unit) and spacing (number of non-stomatal cells between nearest stomata), and in non-stomatal cell shape/size (Table S5).

Figure 8B shows a representative selection of these phenotypes. Overexpression of two R2R3 MYB proteins, MYB74 and MYB34, related to ABA responses (Xin et al., 2005) and IAA synthesis (Celenza et al., 2005), respectively, produced stomatal clusters in otherwise normal seedlings. iMYB74oe also had abnormal non-stomatal cells, with frequent giant rounded cells and small-cell patches in rosette arrays. Recently, ABA was identified as being involved in stomata development (Tanaka et al., 2013), a link that our results with iMYB74oe support. MYB34/ATR regulates the tryptophan pathway and modulates IAA levels, and its transcription is induced by cytokinins (Jones et al., 2010). As it is also involved in indol-glucosinolates homeostasis, MYB34/ATR1 impinges in both primary and secondary metabolism (Malitsky et al., 2008), and stomatal clusters in iMYB34oe might relate to any of these processes. Overexpression of ATHB22, a homeobox-zip TF of unknown function expressed in seedlings and siliques, triggered a strong epidermal phenotype with occasional clusters, frequent unpaired GCs and atypical pavement cell divisions; seedling morphology was normal. The extra cell divisions were not observed if overexpression was induced at 4 dpg, but stomata clusters still formed (not shown), indicating that ATHB22 produces distinct effects in the protodermis and in the developing epidermis. TNY is a NAC-domain protein, whose overexpression renders dwarf plants that accumulate transcripts from DRE- and ERE-driven genes (Sun et al., 2008). A gain-of-function allele with increased *TNY* expression also showed abnormal epidermal cells (Wilson et al., 1996). β-estradiol treatment of iTNYoe induced a very strong phenotype that included severe dwarfism and a very disturbed epidermis, with stomatal clusters and elongated, un-lobed non-stomatal cells.

These and similar results with other TRANSPLANTA overexpressing lines are the first indication that TFs selected by their differential expression in stomataless mutants may be novel players in stomatal and epidermal development gene circuits.

## Conclusions

1. Transcriptomic analysis of expanded cotyledons from two Arabidopsis mutants lacking stomata identified sets of genes commonly regulated in both mutants compared to the wild-type, as well as genotype-specific DE genes. Each genotype expressed diagnostic genes related to stomata development and/or restricted to specific cell stages that matched its distinctive epidermal phenotype. The microarray data (confirmed by qPCR) showed that low abundance, genotype-specific transcripts were detectable in our samples. Hence, our unbiased transcriptomic analysis should also identify novel genes with the potential for explaining the developmental and physiological features of stomataless plants.
2. A set of genes commonly down-regulated in *spch-3* and *mute-3* suggest that cotyledons lacking stomata had depressed S, N, and secondary metabolisms. In contrast, only a few photosynthesis-related genes showed significant down-regulation, though they coded for crucial proteins. Chlorophyll fluorescence imaging revealed that the mutants maintained a depressed but not negligible photosynthesis. This, as well as the transcriptomic data suggestive of chloroplast functionality, indicates that stomataless plants can indeed perform some photosynthesis, which may be related to their (limited) capacity for growth in soil.
3. Both mutants expressed genes indicative of potential for growth at a time (21 dpg) when Col-0 cotyledons did not. Several genes differentially up-regulated in *mute-3* were also identified; these included some high-confidence SPCH targets, as well as development-related genes exclusive to this mutant, which are new candidates for exploring stomatal lineage cell types absent in the other two genotypes.
4. A selection of development-related genes DE in one or the two mutants was examined for epidermal phenotypes associated with their loss-of-function, using T-DNA insertion lines. Of the 24 insertion mutant genes examined, one rendered an epidermal phenotype, leading to the identification of *EDA17* as a new candidate gene involved in stomata development. Conditional overexpression of 19 transcription factors that were DE in the mutants induced altered stomatal phenotypes, suggesting functional roles for these novel transcription factors in stomatal or epidermal differentiation.

### Conflict of interest statement

The authors declare that the research was conducted in the absence of any commercial or financial relationships that could be construed as a potential conflict of interest.
