# Supplementary material for: Transcriptional profiles of Arabidopsis stomataless mutants reveal developmental and physiological features of life in the absence of stomata
Source: Front Plant Sci. 2015 Jun 23;6:456. doi: 10.3389/fpls.2015.00456 (PMC4477074; doi:10.3389/fpls.2015.00456)
Supplement: Supplementary file 2 [file Table2.DOCX]

**Table ST2.** Genes selected for qPCR expression analysis. Gene ID and primers used for qPCR are included.

| **Probeset** | **AGI code** | **Protein name** | **Gene symbol** | **Primers (forward, reverse)** | **Product size (bp)** |
| --- | --- | --- | --- | --- | --- |
| 263761_at | At2g21330 | FRUCTOSE-BISPHOSPHATE ALDOLASE 1 | *FBA1* | ttctcgtcgagcagaacatc | 63 |
|  |  |  |  | caacaagtggcaccaaacc |  |
| 251762_at | At3g55800 | SEDOHEPTULOSE-BISPHOSPHATASE | *SBP* | aatggttcctgatgttaaccaga | 114 |
|  |  |  |  | ccaagaggagccacttcaaa |  |
| 245809_at | At1g58440 | SQUALENE EPOXIDASE 1 | *SQE1* | cccttccccaaaacgaat | 66 |
|  |  |  |  | ggaaatattatccccgatgct |  |
| 258484_at | At3g02580 | DWARF 7 | *DWF7* | cgccggaaccctactatactt | 108 |
|  |  |  |  | gcctttattgtaggaattgcatct |  |
| 251881_at | At3g54250 | Mevalonate diphosphate decarboxylase, putative |  | ctgaagggacaccacaggtt | 65 |
|  |  |  |  | gcgtgcaatcagtacagca |  |
| 258037_at | At3g21230 | 4-COUMARATE:COA LIGASE 5 | *4CL5* | tgatcaaattcaaaggctacca | 112 |
|  |  |  |  | tcagctacttcatccttcattgc |  |
| 252983_at | At4g37980 | ELICITOR-ACTIVATED GENE 3-1 | *ELI3-1* | tttatccatatgggaaaggttctt | 125 |
|  |  |  |  | ttgaacctcacatccttttcac |  |
| 258047_at | At3g21240 | 4-COUMARATE:COA LIGASE 2 | *4CL2* | cggttaggatggttaagtctgg | 112 |
|  |  |  |  | gcttctgtcatcccatagcc |  |
| 251304_at | At3g61990 | O-MTASE FAMILY 3 PROTEIN | *OMTF3* | ccggagtgtctcacaaggtc | 73 |
|  |  |  |  | ccgttctgaatcatagacatcaa |  |
| 253277_at | At4g34230 | CINNAMYL ALCOHOL DEHYDROGENASE 5 | *CAD5* | tgtctctgcttaagcttgatgg | 110 |
|  |  |  |  | gctccccgttatcactttcctc |  |
| 255220_at | At4g05320 | UBIQUITIN 10 | *UBQ10* | cacactccacttggtcttgcgt | 71 |
|  |  |  |  | tggtctttccggtgagagtctt |  |
| 257749_at | At3g18780 | ACTIN 2 | *ACT2* | tcttccgctctttctttccaagc | 77 |
|  |  |  |  | accattgtcacacacgattggttg |  |
