# Supplementary figures and images for: Transcriptional profiles of Arabidopsis stomataless mutants reveal developmental and physiological features of life in the absence of stomata
Source: Front Plant Sci. 2015 Jun 23;6:456. doi: 10.3389/fpls.2015.00456 (PMC4477074; doi:10.3389/fpls.2015.00456)

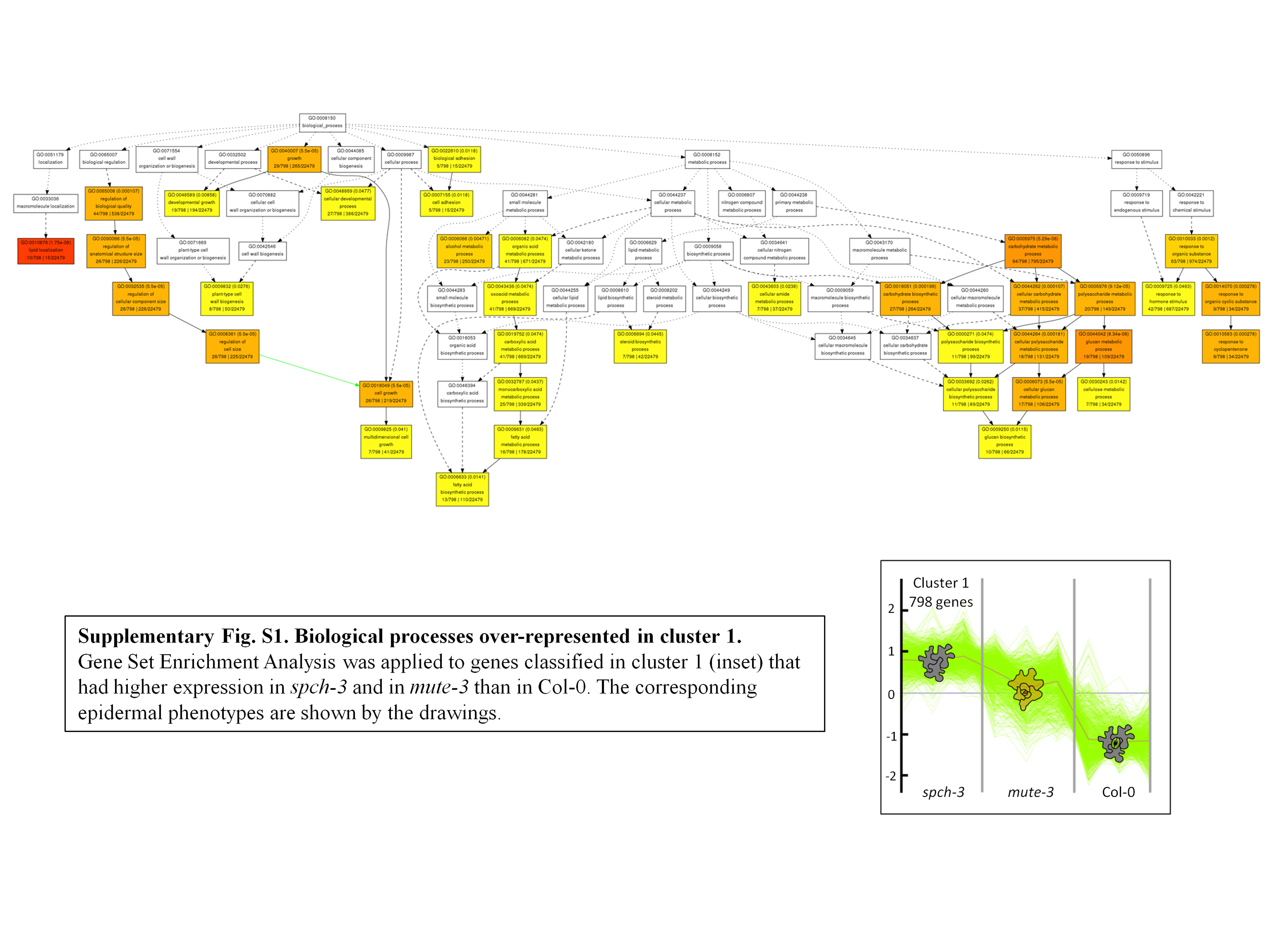

Supplement: Supplementary file 6 [file Image1.TIF]

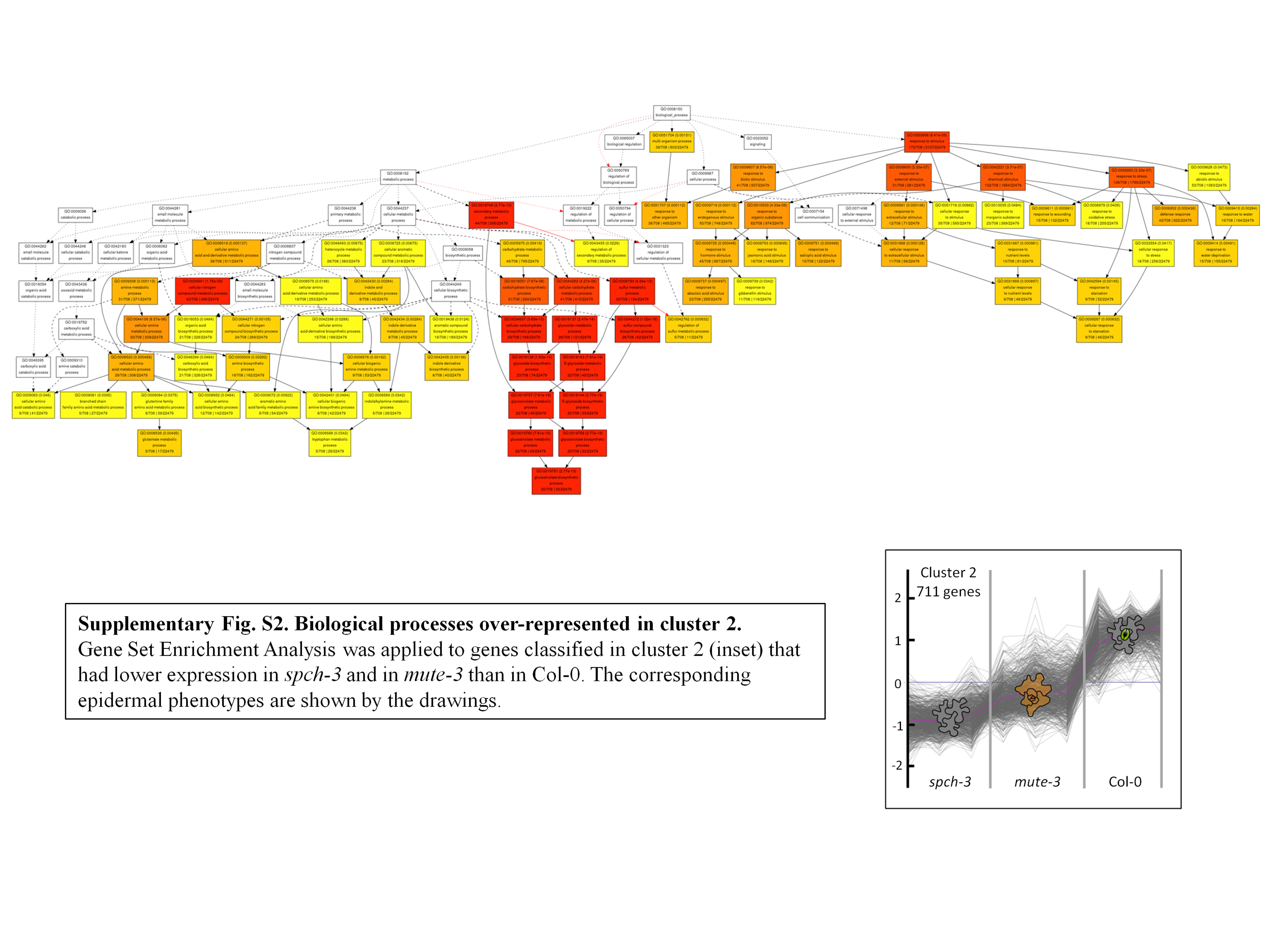

Supplement: Supplementary file 7 [file Image2.TIF]

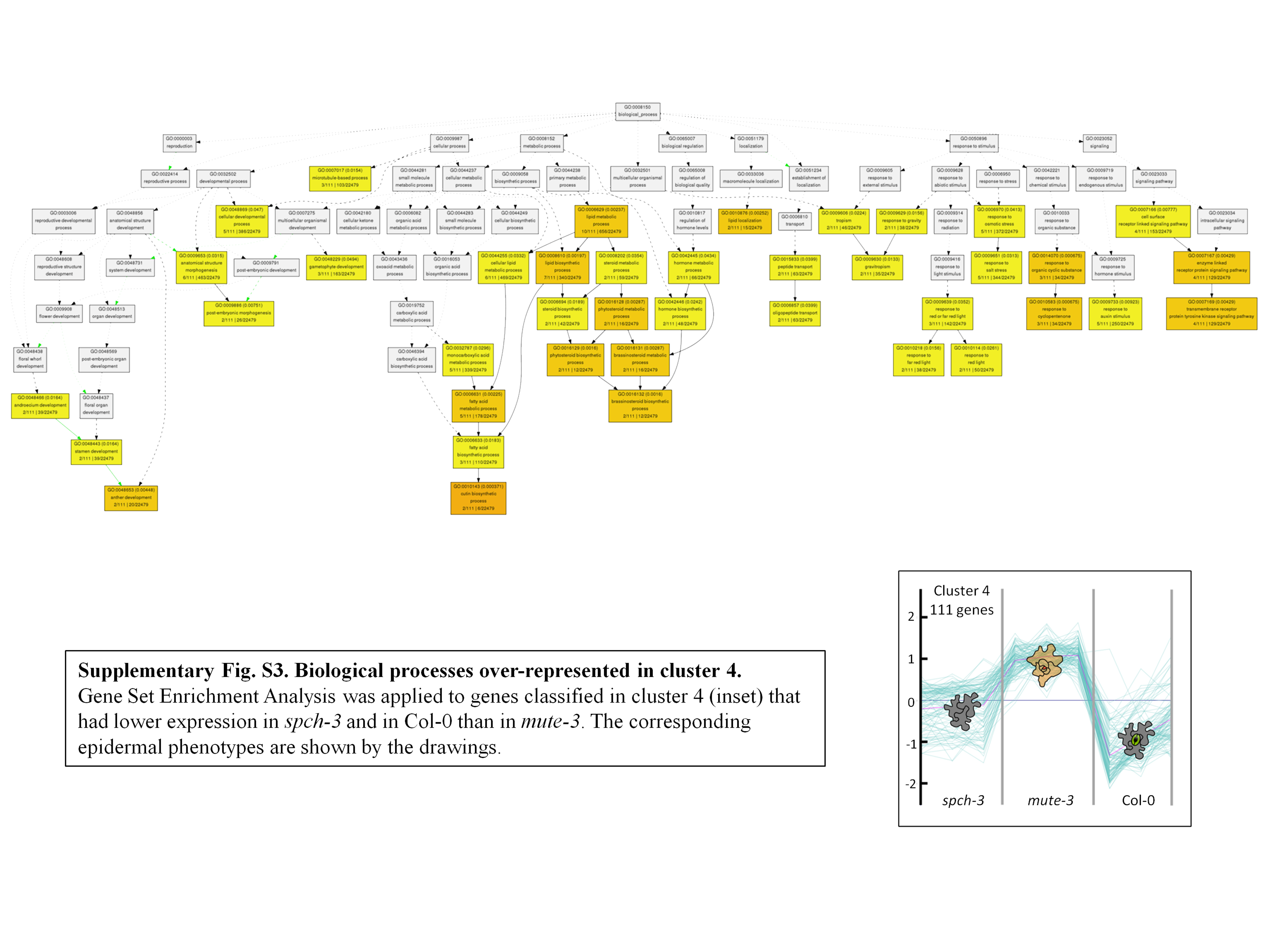

Supplement: Supplementary file 8 [file Image3.TIF]
